# Association between Psoriasis and Chronic Obstructive Pulmonary Disease: A Systematic Review and Meta-analysis

**DOI:** 10.1371/journal.pone.0145221

**Published:** 2015-12-23

**Authors:** Xin Li, Lingjun Kong, Fulun Li, Chen Chen, Rong Xu, Hongshen Wang, Shiguang Peng, Min Zhou, Bin Li

**Affiliations:** 1 Department of Dermatology, Yueyang Hospital of Integrated Traditional Chinese and Western Medicine, Shanghai University of Traditional Chinese Medicine, Shanghai 200437, China; 2 Departmentof Pharmacology& Experimental Therapeutics, Boston University School of Medicine, Boston, MA 02118, United States of America; 3 Research Institute of Tuina, Shanghai Academy of Traditional Chinese Medicine, Shanghai 201203, China; New York University School of Medicine, UNITED STATES

## Abstract

Psoriasis is considered a systemic inflammatory disorder. Previous studies have reported conflicting positive or negative correlations between psoriasis and chronic obstructive pulmonary disease. We performed a meta-analysis to determine whether there is an associated risk between psoriasis and chronic obstructive pulmonary disease. We performed a complete 30-year literature search of MEDLINE, Embase, and Cochrane Central Register databases on this topic. Four observational studies with a total of 13,418 subjects were identified. The odds ratios of chronic obstructive pulmonary disease in subjects with psoriasis/mild-to-moderate psoriasis were analyzed using the random-effects model, while the odds ratios of chronic obstructive pulmonary disease in subjects with severe psoriasis and current smoking in subjects with psoriasis were analyzed using the fixed-effect model. We found that psoriasis patients were at a greater risk of developing chronic obstructive pulmonary disease than the general population (odds ratio, 1.90; 95% confidence interval, 1.36–2.65) and that the association between of psoriasis and with chronic obstructive pulmonary disease was stronger among patients with severe psoriasis (odds ratio, 2.15; 95% confidence interval, 1.26–3.67). Psoriasis patients should be advised to cease smoking to reduce their risk of COPD. Moreover, identification of this potential risk may enable earlier implementation of preventive measures for reduction comorbidity and mortality rates.

## Introduction

Psoriasis is a common chronic and relapsing immune-mediated inflammatory disease of the skin that affects approximately 2–4% of the population worldwide[1]. The clinical phenotype of psoriasis may present with several forms, including plaque, guttate, pustular, and erythrodermic. Psoriasis is characterized by scaly and erythematous patches, papules, and plaques that can be pruritic, which may result in interrupted sleep, impaired concentration, and an overall reduced quality of life[2]. Although the pathogenesis of psoriasis is not completely understood, a re-evaluation of the recent literature indicated that it is a systemic chronic inflammatory disorder[3]. Since various inflammatory autoimmune diseases result from dysregulation of multiple cytokine pathways[4] including inflammatory cytokines that play key roles across the inflammatory diseases, a variety of disease states could be associated with multiple similar systemic inflammatory cascades[5].

Chronic obstructive pulmonary disease (COPD), which encompasses chronic obstructive bronchitis and emphysema, affects approximately 10% of the general population[6]. A progressive but not fully reversible airflow limitation and an inflammatory response in the affected lungs leading to dyspnea and other comorbidities characterizes COPD. While COPD is a preventable and treatable but not currently curable disease, a variety of factors associated with an enhanced chronic inflammatory response have been implicated in its pathogenesis, including immune regulation defects, genetic susceptibility, infection, and environmental factors[7]. Smoking being the most important environmental risk factor and key cause of development of COPD[8, 9], the pathogenesis cannot be strictly attributed to a single compound since cigarette smoke contains thousands of injurious agents[10]. Alveolar destruction and airway remodeling results from exposure to chronic cigarette smoke, bombardment by endogenous mediators of inflammation and cell injury[9].

It is widely accepted that common pathogenic mechanisms are shared among many human chronic inflammatory diseases of unrelated pathology and manifestation. Increasing our understanding of the strength of the correlation between psoriasis and COPD will help ensure that future observational studies include adequate adjustments for the presence of COPD among patients with psoriasis. The purpose of this review was to examine the association between psoriasis and COPD using a meta-analysis.

## Materials and Methods

### Trial Registration

The review protocol was registered in the PROSPERO database before the start of the review process (CRD42015025224).

### Data sources and searches

To identify relevant psoriasis studies that included COPD as an outcome measure, three reviewers (X.L., L.J.K., and F.L.L.) systematically searched the MEDLINE, Embase, and Cochrane Central Register databases using the search terms psoriasis, COPD, and chronic obstructive pulmonary disease. Papers published in English and dated between January 1980 and December 2014were included in this study.

### Study selection

To determine eligibility for inclusion in this review, we screened abstracts using the criteria of case-control, cross-sectional, cohort, or nested case-control design studies examining COPD in relation to psoriasis with no limits on participant age, sex, or nationality. The selection criteria for inclusion were as follows: (i) human-only studies; (ii) provision of original data; (iii) inclusion of a reference group; (iv) provision of odds ratios (ORs), risk ratios, or hazard ratio estimates with confidence intervals (CIs) (or enough data to calculate them); or consideration of COPD as a specific outcome event.

**Fig 1:**
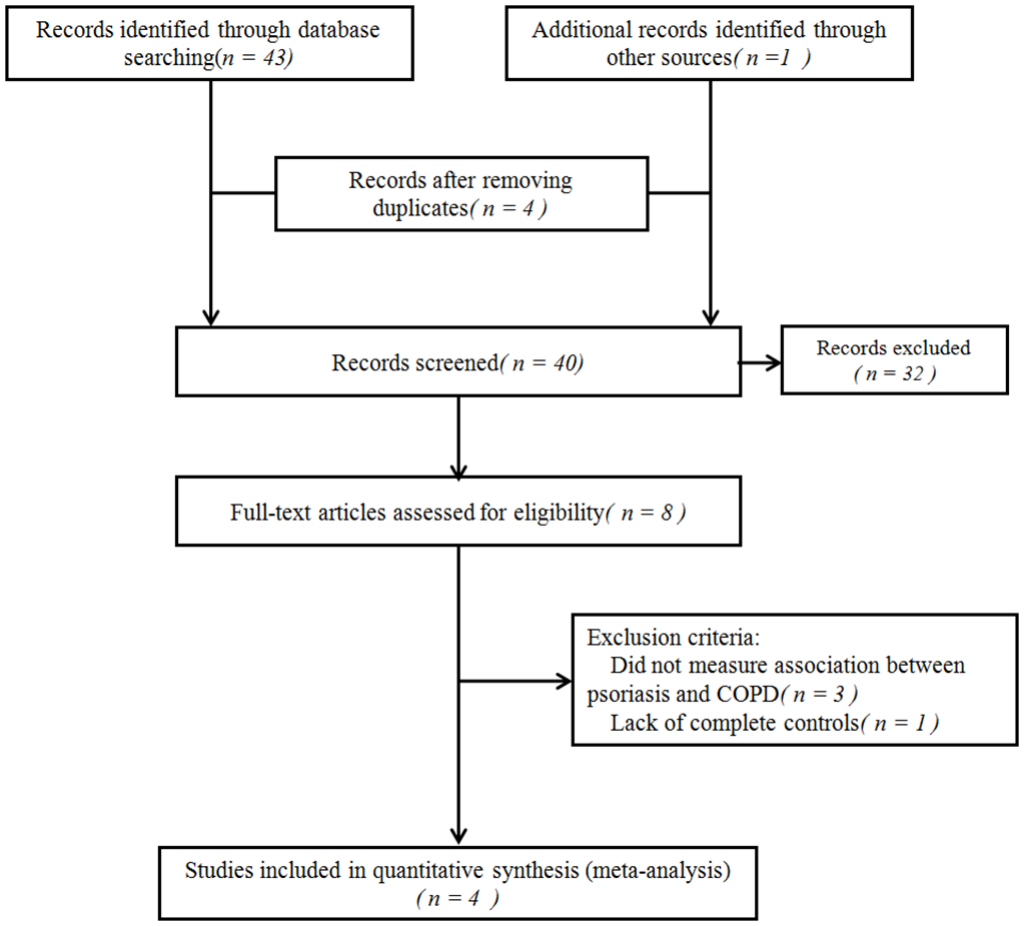
Literature search and study selection.

In this study, we identified 43 articles from the initial search (Fig 1) and through a manual review of the citations from these articles, we found one additional article. After removing four duplicate articles and reading 40 individual abstracts, we identified eight original studies that were eligible for inclusion using the assessment criteria. After a full-text review of these eight studies, we excluded three that did not measure the association between psoriasis and COPD or completely lacked controls. We ultimately selected four studies that met the inclusion criteria for this systematic review[11–14]. The search process is summarized in the flowchart shown in Fig 1.

### Data extraction and quality assessment

Three reviewers independently collected the following descriptive data for each included study: (i) first author; (ii) study characteristics (year, duration, country, setting, design); (iii) participant characteristics (mean age, numbers of cases and controls, number of patients receiving systemic therapy for psoriasis or COPD, smoking status); and (iv) outcome characteristics (diagnostic criteria of psoriasis).

The Newcastle-Ottawa Scale[15] was used to assess study quality by categorizing it into three dimensions: selection, comparability, and exposure for case-control studies; and selection, comparability, and outcome for cohort studies. The selection, comparability, and exposure dimensions contain four, two, and three items, respectively. A star system was used as a semi quantitative assessment of the study quality. A study was awarded a maximum of one star for each numbered item within the selection and exposure categories. A maximum of two stars were awarded for comparability. The number of stars ranged from zero to nine (high-quality, ≥7 stars; medium-quality, 4–6 stars; poor-quality, < 4 stars).

### Data synthesis and analysis

The primary outcome was the association of psoriasis with the risk of COPD for each study. The degree of heterogeneity between studies was assessed using I^2^ tests. An I^2^ value > 50% was considered as substantial heterogeneity, and DerSimonian and Laird random-effect models were used to compute the globalOR. However, if the inter-study heterogeneity was not substantial (I^2^< 50%), a fixed-effect model was considered suitable. To investigate possible reasons for heterogeneity, we performed subgroup analysis and meta-regression using pre-specified variables and random-effects meta-analysis. Pre-specified sources of heterogeneity in meta-regression included study location, source population, study design, study quality, severity of psoriasis, psoriatic arthritis included and outcome ascertainment. The methods and findings of this review strictly followed the Meta-analysis of Observational Studies in Epidemiology group guidelines and checklist[16]. The Cochrane Collaboration software Review Manager 5.2 was used for meta-analysis (http://ims.cochrane.org/revman). Meta-regression was performed using STATA version 10.0 (STATA Corp, College Station, TX, USA.).

## Results

Of a total of 44 studies, four observational studies conducted in Europe (one study), Middle East (two studies), and East Asia (one study) in inpatient and outpatient settings, were selected through a systematic review and included in this meta-analysis[11–14]. The four studies included 13,418 participants (3,241 patients with psoriasis, 10,177 controls) who met the inclusion criteria for the dichotomous variables of the systematic review (Table 1). COPD was considered a specific dichotomous outcome event and ORs were provided in all four studies. Three studies reported a statistically significant difference in the incidence of COPD between psoriasis patients and controls[11, 13], and all included data on the use of systemic psoriasis treatments[11–13]. More specifically, patients with severe psoriasis received systemic therapy (e.g. phototherapy, psoralen plus ultraviolet A therapy, systemic therapies, or a combination there of), while those with mild psoriasis received topical medication. While two studies included data on COPD drug treatment[12, 13], the other two reported smoking status[12, 14]. Newcastle-Ottawa Scale scores of 4–9 are shown in Table 2. Two studies were deemed of medium quality (4–6stars), while the other two were deemed of high quality (7 or >7stars).

**Table 1:** Included Observational Studies.

Author (pub. year)	Study setting	Study Period MM/YY-MM/YY	Study design	Outcome	Controls: total number and number with COPD (%)	Cases: total number and number with COPD (%)	Mean age of controls, years, mean (SD)	Mean age of cases, years, mean (SD)	Cases receiving systemic therapy psoriasis	Use of COPD drugs (%)	Smoking status (%)
Chiang 2012	Taiwan; NR (LHID2005 and NHID)	01/2004-12/2005	Retrospective cohortstudy	Psoriasis (ICD-9-CM codes 696.0, 696.1, and 696.8); COPD (ICD-9-CM codes 491, 492 and 496)	Total: 8342, COPD: 42(0.05)	Total: 2071, COPD: 25(1.21); Mild psoriasis: 1580, COPD 18(1.14); Severe psoriasis 491, COPD 7(1.43)	Matched with patients group in terms of age (<30, 31–40, 41–50, 51–60, 61–70, and >70 years)	NR	Severe-psoriasis received systemic therapy (including phototherapy); mild-psoriasis received topical medication	NR	NR
Wakkee 2011	Netherlands; outpatient/inpatient (PHARMO Record Linkage System)	1997–2008	Retrospective cohort study	International Classification of Diseases, Ninth Revision, Clinical Modification	Total: 128710, COPD: 13379(10.40)	Total: 25742, COPD: 5834(22.70)	38.2(22.9)	44.3(19.6)	Severe psoriasis received systemic therapy (i.e., PUVA therapy, systemic therapies, inpatient treatment or a combination of these)	Psoriasis cohort: 22.7; Reference cohort: 10.4	NR
Al-Mutairi 2010	Kuwait; outpatient	01/ 2003-12/2007	Retrospective case-control study	NR	Total: 1835, COPD: 74(4.03)	Total: 1835, COPD: 98(5.34); Mild-moderate psoriasis: 1661, COPD 89(5.36); Severe psoriasis 129, COPD 9(6.98)	52.7(13.5)	52.3(11.9)	Psoriasis received significantly wider varieties of systemic drugs	Psoriasis: 4.3; Control: 3.8	Current smoker Psoriasis: 51.34; Control: 32.5; Ex-smoker Psoriasis: 22.98; Control: 15.51
Dreiher 2008	Israel; NR (CHS)	NR	Retrospective case-control study	The diagnoses of COPD was taken from the CHS chronic diseases registry	Total: 24287, COPD: 873(3.60)	Total: 12502, COPD: 716(5.70)	54.3(17.5)	55.8(16.7)	NR	NR	Current smoker Psoriasis: 12.5; Control: 9.6

LHID, longitudinal health insurance database; NHID, national health insurance database; NR, not reported; PUVA, psoralen plus ultraviolet A

**Table 2:** Newcastle–Ottawa Scale (NOS) Quality Assessment Table.

Study	Selection	Comparability	Exposure/outcome	Overall star rating
Chiang 2012	++++	++	++	8
Wakkee 2011	+++		++	5
Al-Mutairi 2010	+++	++	++	7
Dreiher 2008	++++		++	6

A star system was used for allow a semi quantitative assessment of study quality. A study was awarded a maximum of one star for each numbered item within the selection and exposure categories. A maximum of two stars were awarded for comparability. The NOS ranges from zero to nine stars. We considered high-quality studies as those that achieved seven or more stars, medium-quality studies those with four to six stars, and poor-quality studies those with fewer than four stars.

**Fig 2:**
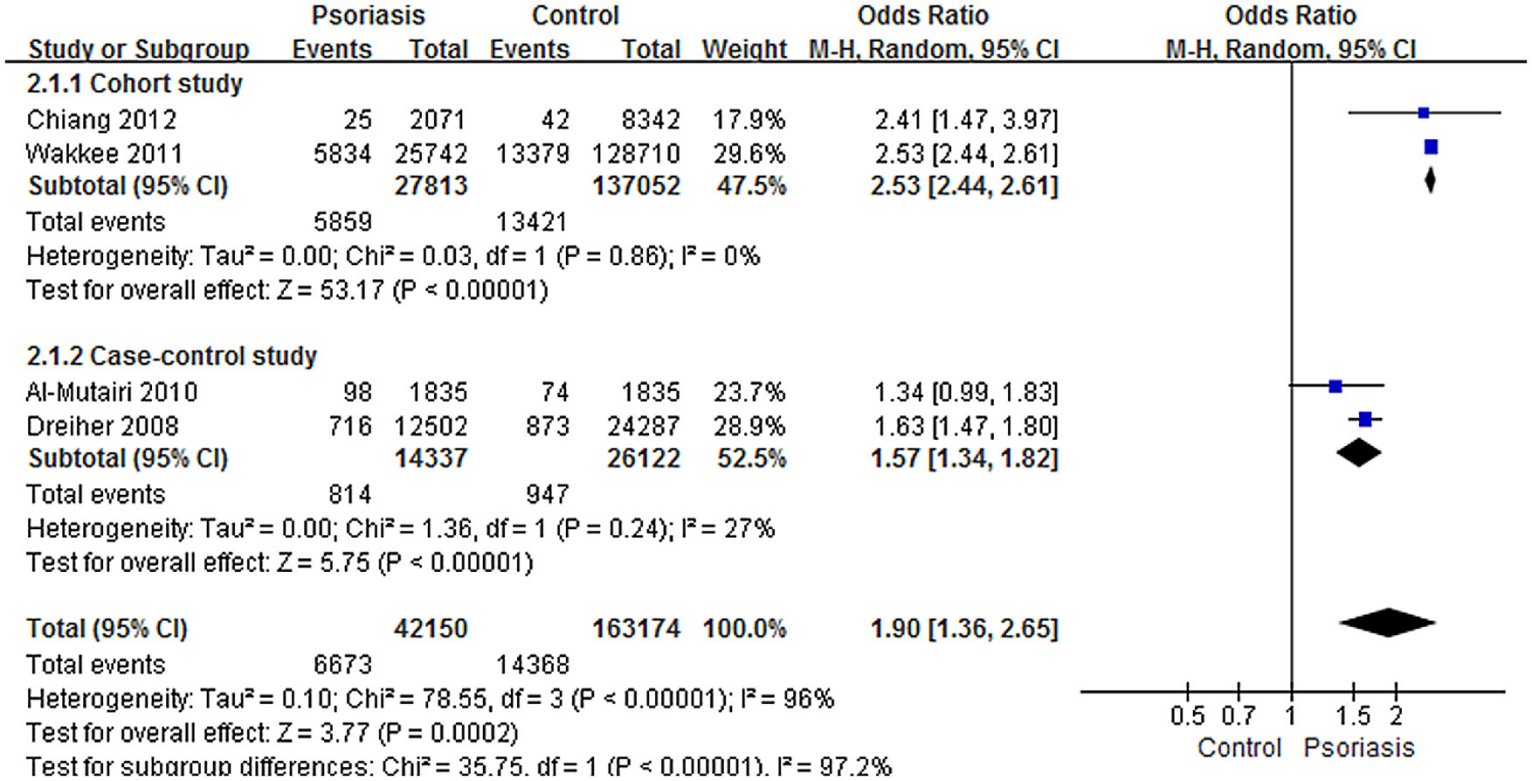
Meta-analysis of the prevalence of chronic obstructive pulmonary disease (COPD) in patients with psoriasis compared with controls. “Events” means the number of COPD in subjects. Odds ratios (ORs) for COPD in subjects with psoriasis compared with subjects without psoriasis. The point estimate (center of each blue square) and the statistical size (proportional area of the square) are shown. Horizontal lines indicate 95% confidence intervals. The pooled OR (diamond) was calculated using a random effects model.

The meta-analysis of COPD prevalence in patients with or without psoriasis revealed significant inter-study heterogeneity (I^2^ = 96%). However, low heterogeneity within each separate subgroup (Cohort study I^2^ = 0%; Case-control study I^2^ = 27%) were displayed. A random-effects modeling analysis of the pooled data from the four studies showed a significant effect between psoriasis patients and controls on the associated prevalence of COPD (OR, 1.90; 95% CI, 1.36–2.65) (Fig 2). An optimal subgroup for the Cohort study (OR, 2.53; 95% CI, 2.44–2.61) was identified by subgroup analysis. In addition, a significant difference was found between the subgroups from the Case-control study (OR, 1.57; 95% CI, 1.34–1.82). Meta-regression of the association between psoriasis and COPD did not reveal any statistically significant sources of heterogeneity (Table 3).

**Table 3:** Potential prespecified sources of heterogeneity explored among studies reportingan association between psoriasis and COPD.

Prespecified source of heterogeneity	No. of studies	Random-effects meta-regression (95% CI)	P-value
Study location			0.240
Europe	1	0.025(1.144–2.118)	
Asia	3	0.025(1.069–1.455)	
Other	0	N/A	
Source population			0.454
Inpatient	0	N/A	
Outpatient	1	0.312(0.150–2.704)	
No distinction	3	0.312(0.370–6.665)	
Study design			0.360
Case–control	2	0.013(0.506–0.792)	
Cohort	2	0.013(1.262–1.976)	
Study quality			0.630
High-quality(≥7stars)	2	0.700(0.180–4.023)	
Medium-quality(4–6stars)	2	0.700 (0.249–5.545)	
poor-quality(<4stars)	0	N/A	
Severity of psoriasis			0.690
No distinction	2	0.700 (0.180–4.023)	
Mild vs. severe	2	0.700 (0.249–5.545)	
Psoriatic arthritis included			1.000
No	0	N/A	
Yes	2	0.985(0.193–5.109)	
Not clear	2	0.985(0.196–5.191)	
Outcome ascertainment			0.500
Billing data	1	0.312(0.150–2.704)	
Chart review	3	0.312(0.370–6.665)	
Examination	0	N/A	

CI, confidence interval; N/A, not applicable.

**Fig 3:**
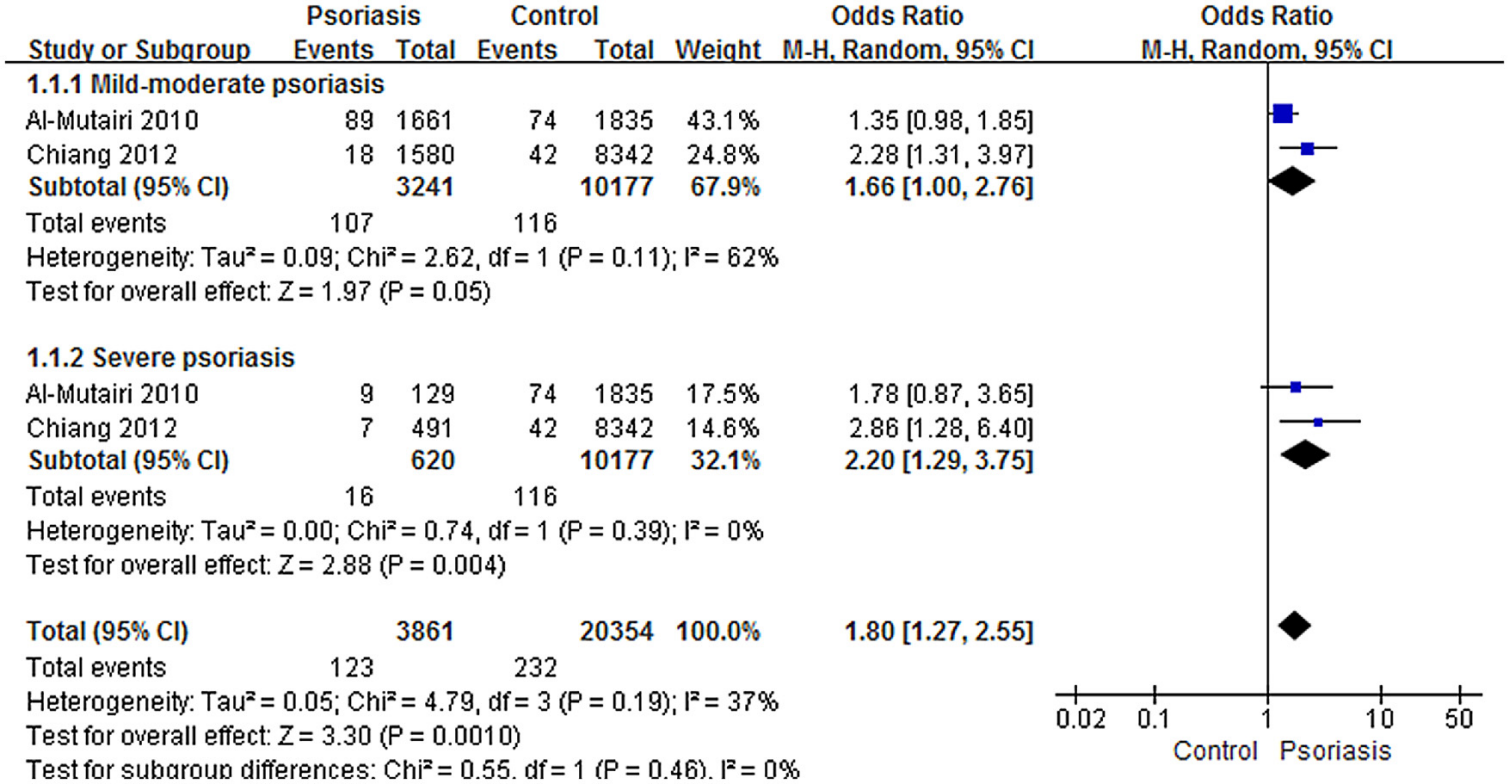
Meta-analysis of the prevalence of chronic obstructive pulmonary disease (COPD) in patients with mild to moderate or severe psoriasis compared with controls. “Events” means the number of COPD in subjects. Odds ratios (ORs) for COPD in subjects with psoriasis compared to subjects without psoriasis. The point estimate (center of each blue square) and the statistical size (proportional area of the square) are shown. Horizontal lines indicate 95% confidence intervals. The pooled ORs (diamond) were calculated using random effects model.

A meta-analysis of COPD prevalence among mild-to-moderate or severe patients with psoriasis and controls revealed moderate or low inter-study heterogeneity, respectively (mild-to-moderate psoriasis, I^2^ = 62%; severe psoriasis, I^2^ = 0%), and a significant effect of the associated prevalence of COPD between severe patients and controls (Fig 3). On Random-effects modeling, a significant effect was observed in two studies[11, 12] with a pooled OR of 2.20 (95% CI, 1.29–3.75). However, no significant effect was found between patients with mild-to-moderate psoriasis and controls using random-effects modeling of the same two studies[11, 12] with a pooled OR of 1.66 (95% CI, 1.00–2.76) (Fig 3).

**Fig 4:**
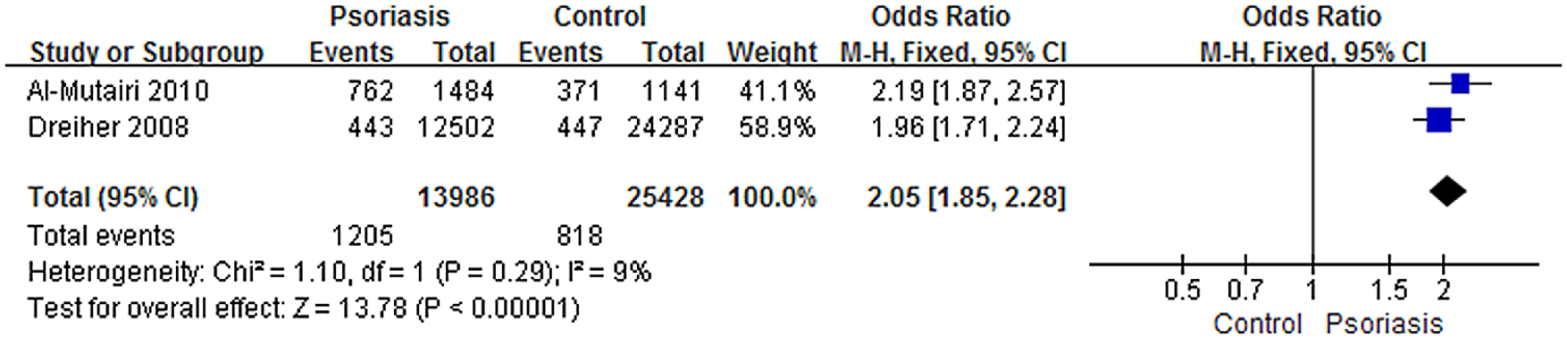
Meta-analysis of psoriasis and prevalence of current smoking compared with controls. “Events” means the number of current smoking in subjects. Odds ratios (ORs) for current smoking in subjects with psoriasis compared with subjects without psoriasis. The point estimate (center of each blue square) and the statistical size (proportional area of the square) are shown. Horizontal lines indicate 95% confidence intervals. The pooled OR (diamond) was calculated using a fixed-effects model.

The association between psoriasis and the prevalence of smoking addressed in two studies[12, 14] showed low inter-study heterogeneity (I^2^ = 9%), while the pooled data showed a significant effect between psoriasis patients and controls in the prevalence of current smoking (OR, 2.05; 95% CI, 1.85–2.28) (Fig 4). Regrettably, we could not confirm a direct association between smoking and COPD in patients with psoriasis.

## Discussion

Our results demonstrated that psoriasis patients are at a greater risk of developing COPD (OR, 1.90; 95% CI, 1.36–2.65), and the association was stronger in patients with severe psoriasis (OR, 2.15; 95% CI, 1.26–3.67).

This study collected and reanalyzed individual participant data including a total of 13,418 participants from four observational studies for the effects of psoriasis on the incidence of COPD. These studies provide almost all of the available epidemiological evidence worldwide on the topic. Since the 1980s, only five studies[11–14, 17] have been conducted to investigate the possible correlation between psoriasis and COPD. A previous study observed a higher prevalence of COPD (5.0%) than expected based on the total U.S. population data (1.7%) in 2005[17]. Moreover, the results of a large, population-based case-control study further supported the association between psoriasis and COPD, and suggested smoking cessation as a COPD risk-reduction strategy in patients with psoriasis[14], although whether the correlation between smoking and COPD events is circumstantial or causal in psoriasis patients remains to be answered. In contrast[12], a case-control study conducted in the Middle East provided conflicting results. Therefore, since the association between psoriasis and COPD remains unclear and controversial among clinicians, a meta-analysis study was needed to further examine the correlation between psoriasis and COPD.

The consistent association between psoriasis and COPD suggests a likely pathophysiologic link between the two diseases. The hypothesis of a common cytokine-based pathology, wherein one inflammatory autoimmune disease significantly increased the risk of another, is gradually being accepted[18]. It is believed that the inflammatory response in psoriasis leads to a Th1 lymphocyte cytokine milieu with increased levels of IL-1, IL-6, IL-8, TNF-α, and markers of systemic inflammation[3, 19]. Similarly, elevated levels of these pro-inflammatory cytokines have also been found in the sputum and bronchoalveolar lavage of COPD patients[20, 21]. Specifically, the T cell receptor and co-stimulatory molecules (e.g. CD3, CD8)[3, 22, 23]; T cell cytokines (e.g. interferon-γ, IL-13, IL-17, IL-23)[3, 21, 24, 25]; other pro-inflammatory cytokines (e.g. TGF-β, IL-12, IL-18,IL-22)[3, 21, 25–28]; chemokines and receptors (e.g. CXCR1, CXCR2, CXCR3)[3, 21, 29]; adhesion molecules (e.g. ICAM-1, E-selectin, CD18)[21, 30, 31]; and proteases (elastase, cathepsins, and matrix metalloproteinases)[21, 32, 33] have all been implicated in the pathogenesis of psoriasis and COPD. These cellular subpopulations and mediators are often excellent targets for monoclonal antibodies or biologics, while several monoclonal antibodies and/or biological reagents have been developed confirm the existence of common molecular targets in clinical studies, including an anti-IL8 antibody (ABX-IL-8)[34], CXCR2 (danirixin)[35], and phosphodiesterase 4 inhibitors (roflumilast)[36, 37], and TNF-α antagonists (etanercept). The fact that similar immunotherapeutic agents have been developed for the treatment of psoriasis and COPD provides further indirect evidence of the close connection between the two diseases.

It is interesting that the pooled OR of the associated prevalence of COPD between the patients with severe psoriasis and controls was significant compared to those with mild-to-moderate psoriasis. Likewise, psoriasis confers an increased risk of developing cardiovascular and ischemic heart disease; only severe psoriasis has been reported as an independent cardiovascular risk factor[38]. Unsurprisingly, cigarette smoking, the most important environmental risk factor involved in the development of COPD[8], has been proposed as an independent risk factor for the development of psoriasis[39]; this is also supported by the findings of the present study.

This study has several strengths. We performed a careful meta-analysis using three different data sources. Multiple authors reviewed the data independently, thereby minimizing the risk of publication bias or missing data. By separating prevalence in the analysis, as well as the severity of psoriasis, we were able to better quantify the possible temporal association between psoriasis and COPD. We also performed meta-regression analyses to identify sources of heterogeneity that commonly occur during meta-analyses of observational data. While conducting this study, we were fully aware of several well-documented limitations, including the distorting effects that publication and location bias may have on systematic reviews and meta-analyses[40]. Only four observational studies from Europe, Middle East, and East Asia were eligible and reviewed, although we were confident that our search strategy located all relevant studies, a certain degree of uncertainty persisted. As the included studies did not test the causation, we could not infer a causal relationship between psoriasis and COPD according to the available evidence from primary studies. As all systematic reviews must be interpreted in the context of the available primary studies, the variations in study findings observed here are likely attributable to differences in study design, patient population, and the ascertainment of predictors and outcomes. Ethnic-specific differences in COPD prevalence and severity were identified among genotypes as well as other factors such as genetic–environmental interactions [41].

Meanwhile, COPD is more prevalent in patients of middle and older age: the mean age of participants from the three included studies was 44.3 years, which almost coincides with the COPD onset age[12–14]. However, despite the age- and ethnicity-related factors, the potential heterogeneity among studies and the lack of a significant effect between patients with mild to moderate psoriasis and controls (OR, 1.66; 95% CI, 1.00–2.76), the directionality of the association between psoriasis and COPD were established. Therefore, patients with psoriasis should receive appropriate information regarding this association and should be advised to cease smoking to reduce their risk of COPD. Future studies to infer a causal relationship should include prospective follow-up studies to explore the mechanisms underlying the association between the two conditions and investigate the role of systemic psoriasis therapies for preventing COPD.

## Conclusions

In summary, patients with psoriasis are at greater risk of developing COPD. The association between psoriasis and COPD was stronger in patients with severe psoriasis. Physicians should be aware of this potential risk to reduce comorbidity and mortality rates. Evidence of the link between psoriasis and COPD is strengthened by the fact that a number of monoclonal antibodies and/or biological reagents currently under clinical development have been designed for use in patients with psoriasis or COPD.

## Supporting Information

S1 MOOSE Checklist MOOSE checklist. (DOC)
