# Supplementary material for: Association between Psoriasis and Chronic Obstructive Pulmonary Disease: A Systematic Review and Meta-analysis
Source: PLoS One. 2015 Dec 23;10(12):e0145221. doi: 10.1371/journal.pone.0145221 (PMC4689442; doi:10.1371/journal.pone.0145221)
Supplement: S1 MOOSE Checklist — (DOC) [file pone.0145221.s001.doc]

**MOOSE Checklist**

**Association between Psoriasis and Chronic Obstructive Pulmonary Disease: A Systematic Review**

Xin Li, M.D.^1,2^, Lingjun Kong^3^, M.D.^1^, Fulun Li, M.D., Ph.D.^1^, Chen Chen, Ph.D.^2^, Rong Xu, M.D.^1^, Hongshen Wang, M.D.^2^, Shiguang Peng, M.D.^2^, Min Zhou, M.D.^1^, Bin Li, M.D., Ph.D.^1*^

^1^Department of Dermatology, Yueyang Hospital of Integrated Traditional Chinese and Western Medicine, Shanghai University of Traditional Chinese Medicine, Shanghai 200437, China

^2^Departmentof Pharmacology& Experimental Therapeutics, Boston University School of Medicine, Boston, MA 02118, USA

^3^Research Institute of Tuina, Shanghai Academy of Traditional Chinese Medicine, Shanghai 201203, China

* Corresponding author:

Bin Li

Address: No.110, Ganhe Rd. Shanghai, China 200437

Tel: +86 02165161782

Fax: +8602165164621

Email: 18930568129@163.com

| **Criteria** | | **Brief description of how the criteria were handled in the meta-analysis** |
| --- | --- | --- |
| **Reporting of background should include** | |  |
| √ | Problem definition | Psoriasis is a common, chronic, relapsing immune-mediated inflammatory disease. Psoriasis prevalence is on the rise, while an association between psoriasis and chronic obstructive pulmonary disease (COPD) is suggested from previous studies, but results are conflicting. |
| √ | Hypothesis statement | Patients with psoriasis were at a greater risk of developing COPD. |
| √ | Description of study outcomes | COPD |
| √ | Type of exposure or intervention used | Psoriasis |
| √ | Type of study designs used | Observational (case-control, cross-sectional, cohort or nested case-control) studies that compared the prevalence or incidence of COPD among patients with psoriasis with individuals serving as controls. |
| √ | Study population | We placed no restriction. |
| **Reporting of search strategy should include** | |  |
| √ | Qualifications of searchers | The credentials of the three investigators X. Li, L. Kong and F. Li are indicated in the author list. |
| √ | Search strategy, including time period included in the synthesis and keywords | Time period: from January 1980 to December 2014.  Keywords: psoriasis, COPD and chronic obstructive pulmonary disease. |
| √ | Databases and registries searched | MEDLINE, Embase, and Cochrane Central Register databases. |
| √ | Search software used, name and version, including special features | We did not employ a search software. EndNote was used to merge retrieved citations and eliminate duplications. |
| √ | Use of hand searching | We hand-searched bibliographies of retrieved papers for additional references. |
| √ | List of citations located and those excluded, including justifications | Details of the literature search process are outlined in the flow chart. The citation list is available upon request. |
| √ | Method of addressing articles published in languages other than English | We limited to the studies published in English. |
| √ | Method of handling abstracts and unpublished studies | We did not contacted authors for abstracts and unpublished studies on the associations of psoriasis with COPD. |
| √ | Description of any contact with authors | Authors were not contacted, as adequate information for the performance of this review was available from studies and abstracts. |
| **Reporting of methods should include** | |  |
| √ | Description of relevance or appropriateness of studies assembled for assessing the hypothesis to be tested | Detailed inclusion and exclusion criteria were described in the study selection section. |
| √ | Rationale for the selection and coding of data | Data extracted from each of the studies were relevant to the population characteristics, study design, exposure, outcome, and possible effect modifiers of the association. |
| √ | Assessment of confounding | Not applicable. |
| √ | Assessment of study quality, including blinding of quality assessors; stratification or regression on possible predictors of study results | The Newcastle-Ottawa Scale was used to assess the study quality, by categorizing it into three dimensions: selection, comparability and exposure for case-control studies; and selection, comparability and outcome for cohort studies. |
| √ | Assessment of heterogeneity | Heterogeneity of the studies were explored within two types of study designs using Cochrane’s Q test of heterogeneity and I^2^ statistic that provides the relative amount of variance of the summary effect due to the between-study heterogeneity. |
| √ | Description of statistical methods in sufficient detail to be replicated | Description of methods of meta-analyses was detailed in the data synthesis and analysis section. |
| √ | Provision of appropriate tables and graphics | We included 1 flow chart, 2 summary tables and 4 figures. |
| **Reporting of results should include** | |  |
| √ | Graph summarizing individual study estimates and overall estimate | Figure 2,3,4 |
| √ | Table giving descriptive information for each study included | Table 1 |
| √ | Results of sensitivity testing | Mild-moderate/severe psoriasis subgroup analysis was performed; effect on heterogeneity is discussed in the paper. |
| √ | Indication of statistical uncertainty of findings | 95% confidence intervals were presented with all summary estimates. |
| **Reporting of discussion should include** | |  |
| √ | Quantitative assessment of bias | Assessed using funnel plots (see below) |
| √ | Justification for exclusion | Papers were excluded on the basis of exclusion criteria listed. We did not systematically exclude any studies on the basis of language or study population size. |
| √ | Assessment of quality of included studies | The Newcastle-Ottawa Scale was used to assess the study quality. Details are outlined in Table 2. |
| **Reporting of conclusions should include** | |  |
| √ | Consideration of alternative explanations for observed results | We discussed the limitations of this study. Only four observational studies from Middle East, East Asia and Europe were eligible and reviewed and we could not infer a causal relationship between psoriasis and COPD. We noted that prospective follow-up studies should be conducted to explore the mechanisms underlying the association between these two conditions and to investigate the role of systemic psoriasis therapies in COPD prevention. |
| √ | Generalization of the conclusions | Patients with psoriasis were at a greater risk of developing COPD. The association of psoriasis with COPD was stronger among patients with severe psoriasis.  Evidence of the link between psoriasis and COPD is strengthened by the fact that a number of monoclonal antibodies and/or biological reagents currently under clinical development have been designed for use in patients with psoriasis or COPD. |
| √ | Guidelines for future research | We recommend future studies on the causal relationship between psoriasis and COPD. |
| √ | Disclosure of funding source | Description of the detail in the funding source section. |

Funnel plot of log OR for included studies. The approximate symmetry of both plots suggested the absence of significant publication bias affecting studies included in this review. But more clinical trials are needed.
